# Dynamic Comparison of Physicians’ Interaction Style with Electronic Health Records in Primary Care Settings

**DOI:** 10.4172/2329-9126.1000137

**Published:** 2013-12-10

**Authors:** Onur Asan, Jie Xu, Enid Montague

**Affiliations:** 1 Center for Patient Care and Outcomes Research, Division of General Internal Medicine, Medical College of Wisconsin, Milwaukee, WI, USA; 2 Industrial and System Engineering, University of Wisconsin-Madison, Madison, USA; 3 Division of General Internal Medicine and Geriatrics, Feinberg School of Medicine, Northwestern University, Chicago IL, USA

**Keywords:** EHRs, Patient centered care, Doctor-patient communication, Primary care

## Abstract

Researchers have been increasingly interested in the influence of computers on physician-patient communication in consultation rooms because of the substantial growth in the use of Electronic Health Records (EHRs) in the U.S. Previous research showed that physicians have different ways of interacting with patients and EHRs; and these styles may relate to different patterns of nonverbal interaction between the physicians and patients and influence the outcomes of the clinical visit. The purpose of this study was to identify the differences of eye gaze patterns in three EHR interaction styles: the technology-centered style, the human-centered style, and the mixed interaction style. 100 primary care visits with different interaction styles were videotaped. Eye gaze behaviors were coded and described as frequencies and durations of gaze. The dynamic eye gaze patterns of the physicians and patients, in terms of how their gaze behaviors were sequentially associated, were analyzed using lag-sequential analysis. The results indicated that technology-centered group had significantly shorter amount of mutual gaze than other two groups (p=0.032; p=0.015, respectively). In addition, in technology centered style, the physicians were more likely to shift their gaze to the computer when the patients gazed at them; and when the physicians gazed at the computers, the patients were more likely to gaze somewhere else which might be an indicator of disengagement. The study implied that EHRs should be designed in a way that facilitates a positive interaction between the physicians and patients, such as maintaining mutual gaze. Training should also be provided to the physicians for establishing effective and positive interaction styles.

## Introduction

The adoption and use of Electronic Health Records (EHRs) has grown substantially in U.S. medical practices since the deployment of the federal Health Information Technology for Economic and Clinical Health (HITECH) Act [1]. The HITECH Act also instituted financial penalties that will apply if group practices and hospitals do not become meaningful EHR users by 2015 [2], EHR use will likely continue to grow in the next few years. The potential benefits of EHRs in healthcare have been discussed in several studies [3–5]. These benefits include comprehensive documentation of medical records, easy communication with other medical providers, remote access to medical data, and easy access to test results, personal records, health education tools and information resources during medical visits [3–5].

Although there are many potential benefits of EHRs, the presence of computers in the consultation room and the task of computer documentation during medical visits has been shown to cause some unintended consequences which negatively affect physician-patient interaction [6,7]. Studies report that computer use might take the physician’s attention away from the patient [7], cause loss of eye contact with the patient, and direct the physician’s body posture away from the patient [8], thus making patients feel disengaged during the visit [9].

Doctor–patient communication is a fundamental aspect of the health care system; it influences patient outcomes such as satisfaction, trust, compliance, and adherence [10]. Nonverbal communication is a fundamental component of physician-patient communication [11], though it has received limited attention in research compared to verbal communication [12,13]. Furthermore, eye gaze has been reported as the most powerful component of nonverbal communication in a physician-patient encounter [14], and is considered an important activity for establishing common ground and mutual understanding in doctor-patient communication [15]. Studies have explored the duration of doctor gaze at patient [16], patient gaze at doctor [13], and mutual gaze between the doctor and the patient (13) to understand their effect on medical outcomes and patient satisfaction. Gaze has also been used as a variable to analyze doctor-patient-computer interaction in previous studies [9,17].

The effect of computer use on doctor-patient interaction in primary care has been addressed by previous studies [9, 18–20]. One significant finding of the previous research is that physicians employ a variety of different computer-use and communication styles when EHRs are integrated into primary care consultation room [20,21]. Previous studies suggest that the physicians’ specific style of computer use might be the main factor influencing physicians’ communication with patients during medical encounters [22,23]. A recent study reported three distinct EHR interaction styles among physicians, which all had different effects on doctor-patient communication: technologycentered, human-centered, and mixed interaction [23]. Physicians in the technology centered group spent more time typing and gazing at the computer during the visit. The human-centered group spent a minimal amount of time typing, and focused mostly on the patient. The mixed interaction group, on the other hand, shifted their attention and body posture between the patient and the technology throughout the visit [23].

The effect of computer use on doctor-patient communication in primary care was evaluated by using holistic or qualitative measures by previous studies [9,24,25]. However, there is a relative lack of studies which address the effect of computer use on doctor-patient communication dynamically [26]. A previous study used lag sequential analysis to assess eye gaze patterns in primary care encounters where there is no computer in the exam room [26]. Results showed that the patient followed where the clinician was gazing significantly and this occurred relatively soon after the clinician’s gaze behavior. Eye gaze can thus be operationalized as a measure of doctor-patient communication, since it provides an objective and measureable indication of attention and communication [12]. Because eye gaze is such an important and measurable indicator of doctor-patient communication, it is important to understand how eye gaze patterns and durations change when a computer is present in the consultation room, based on physicians’ different EHR interaction styles. Such data can guide the design of EHR technology and training in EHR use to improve clinical outcomes.

The purpose of this study is to compare eye gaze patterns and durations across three groups of physicians with different interaction styles, using lag sequential analysis [23]. Three interaction styles identified by the previous study of this project [23] are compared: technology-centered, human-centered, and mixed interaction. Technology-centered group spent more time typing and gazing at the computer during the visit. Physicians who used a mixed style shifted their attention and body language between their patients and the computer throughout the visit. Human-centered group interacted with the computer less and focused more on the patient. An understanding of eye gaze patterns in different interaction styles in physicians’ work is needed to inform EHR design and training programs that will help physicians effectively integrate EHR use with patient communication.

## Methods

### Study design and sample

The data for this study was derived from a previous study and included one hundred videotaped medical visits [23]. The study was conducted at five Midwestern university primary care clinics. Clinics were identified based on proximity and clinic staff willingness to participate to the study. Physicians and patients were recruited by convenience sampling. Physicians in clinics were invited to the study via invitation letter by the director of the clinic. The criteria for physicians were being a primary care physician and using EHR in their patient care. Residents were not recruited in the study. All patients in the sample had prior relationships with their physician that is they had seen the physicians at least one time prior to the visit. New patients were not recruited to mitigate confounding variables related to interacting with new patient visits. The inclusion criteria for patients were being between 18 and 65 years old, being English speakers, and having less than 30 min scheduled visits. Eligible patients were identified by a member of research team who is affiliated with the clinics and have access to health records, and then eligible patients were invited to the study by a staff of the clinic during the check in process. Patients who agreed to participate were taken to a consultation room which was set up with video cameras. The data was collected between January to June 2011.

Patient group included 56 males and 44 females (mean age: 45.2 years old). Seventy-eight participants were White/Caucasian. Participants have been patients of their primary care doctors for 1–38 years old. Six male and four female physicians participated in the study (mean age: 47.6 years old) and had been practicing family medicine for 5–37 years. The physicians reported using computers in clinical consultations for 3–10 years.

Eye gaze patterns were explored by systematic observations of videotaped medical visits in primary care. Eye gaze patterns and overall interaction in the visit were recorded by using three high-resolutions video cameras from different angles; one camera focused on the patient, one camera focused on the physician, and one captured the whole room to show the overall interaction. The three videos for one visit were synchronized and combined into one video in post processing. Thus there was one video file generated per visit. Informed consent was obtained from both patient and physician participants. The study protocol was approved by university and clinic Institutional Review Boards and HIPAA (Health Insurance Portability and Accountability Act) regulations were fulfilled.

### Data analysis

#### Quantified video coding

First, all videos were coded to get quantified data for descriptive and sequential analysis. Coding can be defined as converting complex data into measurable units [27]. In this study, video coding was used to explore doctor-patient eye gaze patterns and durations during the visit. In order to achieve the goal of the study, a coding scheme was created to record the start, stop, duration, and object of patient gaze and doctor gaze. This coding scheme was modified and validated by previous studies [23,26] (Table 1).

We used the terminology as follows: a) “chart”: documents with information about the patient or notes written by the clinician during the encounter, b) “other artifacts”: objects in the room, including chairs, exam table, sink, medical tools, magazines, etc. c) “Unknown”: the coder could see the subject’s eyes but was not able to specify the object, or the subject gazed at a part of the patient’s body, such as the foot, or back.

Each video (visit) was coded temporally for the entire visit based on the coding scheme. Start and stop times for each code were annotated using a software designed for video coding, evaluation, and analysis. The software calculated the start and stop times, duration, and simultaneous occurrence of two or more codes. Furthermore, we have taken several steps to ensure inter-rater reliability between the coders: all five coders were trained according to the coding scheme, and reliability checks were done regularly to ensure a eliability score above 0.60 Kappa value [28]. A conservative threshold of one sec (meaning any code that deviated by one second or more from another coder was not counted as congruent and thus the reliability was reduced) was used in testing reliability. The average value of Cohen’s Kappa coefficient of all the reliability – check videos was between 0.60 and 0.79. The coders included three undergraduate and two graduate students who were team member of this project and also part of human computer interaction lab of the university.

#### Descriptive analysis

Based on quantified coded data, all eye gaze durations were calculated for each group (technology-centered, human-centered, and mixed) and reported them as a percentage value. These values have been reported as percentage to show the ratio of eye gaze duration to visit length, since visit length might vary per visit. Visit length is defined as the total time the doctor and patient spent together during the visit excluding the physical exam period. Finally, statistical analysis has been used to compare the values across the three groups.

#### Sequential analysis

The purpose of the dynamic analysis was to address the sequence of the occurrence of the eye gaze behavior of the doctor and the patient, in order to investigate how the behavior of the two individuals influences each other. Specifically, lag-sequential analysis helps examine whether one event contributes to the likely occurrence of another [29]. Incorporating the log-linear model, the likelihood ratio chi-square test can be used to test the dependence of initial and response behaviors, whereas adjusted residuals can be used to detect significant behavior sequences [30]. For instance, in this study if “doctor gaze at the computer” (DGT) is the initial behavior and “patient gaze at the computer” (PGT) is the response behavior, we can obtain the frequency of DGT-PGT and the strength of association of these two behaviors by using the lag sequential analysis.

The following procedure was followed to conduct sequential analysis. First, two sets of contingency tables were generated for each group; thus six contingency tables were generated from the data. The first set includes doctor’s behaviors as the initial behavior and patient’s behaviors as the response behavior, and the second set utilizes patient’s behaviors as the initial behavior and doctor’s behaviors as the response behavior. Then, log-linear models were fitted to the contingency tables to examine the dependence of the behaviors as represented by specific cells in the tables. Adjusted residuals were calculated for each cell. If the value of the adjusted residuals in a cell is higher than 2.58, the association of the initial behavior and the response behavior was considered significant; in other words, they were considered to co-occur not by chance [26].

## Results

Statistical tests identified significant differences for eye gaze durations across the groups. Technology withdrawal group had significantly longer visit lengths than other two groups. There is a significant difference in the duration of DGP (doctor gaze at patient) only between the mixed-interaction and technology-centered groups (p=0.021). For mutual gaze, a significant difference between the technology-centered and human-centered groups was obtained (p=0.032), although there was no significant difference for DGP for this pair (p=0.108). Mixed group also had significantly longer mutual gaze than technology centered group (p=0.015). Furthermore, the humancentered group gazed at chart significantly longer than both the technology-centered and mixed-interaction groups (p=0.001). Finally, the technology-centered group typed and gazed at the computer significantly longer than the two other groups [23]. Mean scores of each doctor initiated behavior across the three groups as a percentage of visit length is illustrated in Table 2.

### Sequential analysis

Sequential analysis also yielded some interesting significant patterns for each group. In the human-centered group and mixed-interaction group-, same significant patterns were obtained. For doctors initiated behaviors, the following behavior pairs were significant: DGP-PGD, DGT-PDT, DGC-PGC, DGO-PGO, and DGU-PGU (Tables 3 and 4). Thus, patients responded to doctors’ behaviors by gazing back at the doctor when being gazed at and otherwise following the gaze orientation of the doctor. However, this pattern (PGD-DGP) was not found when the patient’s behaviors were considered initial behaviors. The patient initiated significant behavior pairs included: PGT-DGP, PGC-DGC, PGO-DGO, and PGU-DGU (Tables 3 and 4).

The behavior patterns in the technology-centered group were different from the other two groups. When the doctor’s behavior was considered as the initial behavior, the following behavior pairs were significant: DGP-PGD, DGT-PDT, DGT-PGU, DGC-PGC, DGO-PGO, and DGU-PGU (Table 5). In general, the patients follow the eye gaze orientation of the doctors, which is consistent with the findings of the other interaction styles, as well. However, sometimes when the doctor gazes at the computer, this is followed by patient gaze unknown significantly, which is the unique finding for this group. For patient initiated behaviors, following pairs were significant: PGD-DGT, PGT-DGP, and PGC-DGC (Table 5).

## Discussion

This study used a dynamic approach to compare eye gaze patterns across three different groups of physicians regarding their interaction with EHRs and patients during patient visit. These interaction styles were identified by a previous study [23]. The classification was mainly based on the typing behaviors of physicians, which was the main way of interacting with EHRs, as well as the duration and frequency of doctor and patient gaze at the computer [9, 31]. As the previous study reported, there is a positive relationship between typing and doctor gaze at EHR durations. The descriptive statistics in this study also indicated that there is a reverse relationship between doctor gaze at technology and doctor gaze at patient. The human-centered and mixed-interaction groups gazed at the patient almost half of the visit length during the visit which was significantly longer than technology-centered doctors. Eye contact is an important activity to establish common ground and rapport between physician and patient [13], and our results indicate that technology-centered doctors also have the significantly lowest eye contact (mutual gaze) duration with patients. This might be the byproduct of extensive typing and gazing at EHRs. Our study thus supports some of the earlier studies which found that higher computer use during the visit might cause loss of eye contact between patient and doctor [8,9]. Furthermore, it is also interesting to see that humancentered doctors used paper charts for 13 percent of the visit time in addition to using EHRs. Using paper charts might help them to face to the patient while taking notes, allowing them to maintain eye contact more consistently.

The results of the dynamic analysis showed that the behavior patterns in the human-centered style visits and the mixed-style visits are similar. This similarity is interesting given the fact that the computer usage in these two styles is different. This suggests that the influence of computer usage on how the patient responds to the doctor is moderated by the doctor’s communication style or how the doctor responds to the patient’s behavior. In other words, using technology in the consultation room does not necessarily have a negative effect on doctor-patient communication; the effect depends on how the doctor uses the technology. For instance, the results showed that when the doctor gazed at the EHR, the patient also gazed at the EHR significantly regardless of whether or not the patient can understand or comprehend the information on the screen. This may indicate that doctors can involve patients effectively in the interaction with computers and educate patient with simply sharing the screen to increase their understanding of their own health status. This might eventually improve patient centered care, since screen sharing was suggested as a behavior to improve physician- patient communication with some of the early studies [32]. For instance, a study indicated that when physicians share information visually from the EHR screen, patient satisfaction and their involvement in the decision-making process improves [33].

In the technology-centered style, the behavior pair DGT-PGU was significantly associated, while this pair was not associated in the other two styles. This may indicate that heavy computer use and typing in the technology-centered style leads to the patient’s inattention. This finding thus quantitatively supports the findings of previous qualitative studies, which reported that high computer use might make patients less attentive and disengaged them from communication [9]. This pair DGT-PGU may have potential negative effects on the doctor-patient communication and on patient outcomes. For example, less mutual attention may decrease mutual understanding and negatively affect rapport building between doctor and patient.

Typing is related to the documentation process of physicians during the visit. It is a necessary action for physicians to input information into medical records, and documentation during the visit decreases the time used for charting after hours. However, the documentation and typing style physicians using during the visit might influence patient outcomes, satisfaction, and trust in the physician. Therefore, it is critical for technology designers to understand the importance of nonverbal communication when designing more human-centered technologies such as EHRs. For instance, eye gaze can indicate the focus and level of people’s attention, and body language can indicate their satisfaction and comfort level. Future EHR systems could have improved documentation systems for doctors, so they can document necessary information and also keep patients engaged in communication. In this study, the human-centered and mixedinteraction groups had a good balance of communication patterns, and they did not have the problematic DGT-PGU pattern. However future studies should analyze if these doctors document sufficiently, whether they miss any data, and how many hours they spend in their office for charting so that we can fully understand which communication style is best for both doctor and patient.

## Conclusion

This study explored eye gaze patterns in three different style of physicians interaction with electronic health record systems. The results showed that physician initiated gaze is an essential driver of the interactions between physicians, patient and technology. This finding supports the importance of training physicians for nonverbal communication skills, such as inducing desired gaze behaviors for the patient while using the computers in the consultation room. This study might also assist those creating new forms of technology training for physicians. For example, a training intervention could help clinicians increase gaze at patient in an encounter involving EHRs. This would be an important intervention for future studies because thus far limited information is available on training clinicians to work with clinical information systems [34]. Furthermore, this study also has implications for technology redesign. By understanding the impact that technology has on doctor-patient interactions, future designers can create better EHR systems, which might include new documentation functions. For instance, significant patient –initiated behaviors might indicate a potential opportunity for physicians to share information with using EHRs’ screens.

This study also has some limitations and suggests some possibilities for future work. The sample size of physicians is small; future studies could confirm whether these results are true in a bigger sample of physicians. In addition, this study looked at only one single EHR system, so different systems might yield different results. Future researchers would do well to compare the impacts that different EHR systems have on doctor-patient interaction and inform better HIT design to suggest optimal doctor-patient interaction. Future studies also need to take a longitudinal approach to understand the overall effect of each system on patient outcomes over time.

## Figures and Tables

**Table 1:** Coding scheme used for this study.

Code	Definition
PGD	Patient gaze doctor
PGT	Patient gaze technology (computer)
PGC	Patient gaze chart
PGO	Patient gaze other artifact
PGU	Patient gaze unknown
DGP	Doctor gaze patient
DGT	Doctor gaze technology (computer)
DGC	Doctor gaze chart
DGO	Doctor gaze other artifact
DGU	Doctor gaze unknown

**Table 2:** Mean scores of doctors’ behaviors and visit length.

	Human Centered (n=40 visits)	Technology Centered (n=20 visits)	Mixed (n=40 visits)
**Visit length**	15.4 min.	24.16 min.	16.85 min.
**Doctor gaze at EHR**	24.9 %	49.6 %	34.8 %
**Typing**	2.8 %	21.6 %	8.5 %
**Doctor gaze at chart**	13.8 %	4.30%	4.80 %
**Doctor gaze at patient**	46.7 %	39.49%	48.6 %
**Mutual gaze (Eye contact)**	35.25%	22.44%	34.3 %
**Doctor gaze at unknown**	10.01%	4.77%	5.24 %

**Table 3:** Observed frequencies (conditional probabilities) and adjusted residuals in human-centered style.

**Initial behavior**		**Response behavior**			
	**PGD**	**PGT**	**PGC**	**PGO**	**PGU**
**DGP**	**520(56%) 12.41**	25(3%) −11.69	57(6%) −4.89	24(3%) −2.75	304(33%) −0.54
**DGT**	137(25%) −8.04	**214(39%) 21.95**	25(5%) −4.65	11(2%) −2.60	155(29%) −2.68
**DGC**	124(27%) −6.54	31(7%) −4.07	**130(28%) 15.02**	15(3%) −0.79	157(34%) 0.52
**DGO**	35(32%) −1.84	4(4%) −2.83	6(6%) −1.54	**27(25%) 11.49**	37(34%) 0.14
**DGU**	104(45%) 1.52	7(3%) −4.54	4(2%) −4.33	12(5%) 1.07	**103(45%) 3.89**
	**DGP**	**DGT**	**DGC**	**DGO**	**DGU**
**PGD**	466(43%) −0.63	238(22%) 2.07	210(19%) 0.25	35(3%) −2.80	141(13%) −0.11
**PGT**	**139(54%) 3.73**	58(23%) 1.11	40(16%) −1.49	7(3%) −1.42	12(5%) −4.21
**PGC**	91(46%) 0.69	32(16%) −1.47	**58(29%) 3.80**	12(6%) 1.13	6(3%) −4.40
**PGO**	19(29%) −2.34	10(15%) −0.95	9(14%) −1.09	**15(23%) 7.38**	12(18%) 1.32
**PGU**	249(41%) −1.52	107(18%) −1.81	106(17%) −1.24	30(5%) 0.64	**118(19%) 5.45**

***Note:*** Values in parentheses were adjusted residuals. Highlighted cells showed statistical significance, α<0.01

**Table 4:** Observed frequencies (conditional probabilities) and adjusted residuals in mixed style.

**Initial behavior**	**Response behavior**				
	**PGD**	**PGT**	**PGC**	**PGO**	**PGU**
**DGP**	**578 (52%) 6.51**	47 (4%) −8.27	42 (4%) −2.25	42 (4%) −3.03	404 (36%) 0.28
**DGT**	261 (28%) −5.50	**276 (30%) 13.90**	22 (2%) −3.88	51 (6%) −0.59	314 (34%) −0.92
**DGC**	59 (32%) −1.63	4 (2%) −4.12	**60 (33%) 16.03**	8 (4%) −0.91	53 (29%) −1.59
**DGO**	25 (23%) −2.82	8 (7%) −1.72	2 (2%) −1.59	**40 (36%) 13.01**	35 (32%) −0.70
**DGU**	89 (40%) 0.11	3 (1%) −4.87	10 (5%) −0.53	12 (5%) −0.36	**108 (49%) 3.20**
	**DGP**	**DGT**	**DGC**	**DGO**	**DGU**
**PGD**	492 (42%) −0.34	378 (32%) 0.22	71 (6%) −0.57	64 (5%) −0.23	159 (14%) 0.85
**PGT**	**175 (60%) 4.44**	88 (30%) −0.60	12 (4%) −1.62	9 (3%) −1.86	8 (3%) −4.80
**PGC**	57 (44%) 0.16	26 (20%) −2.44	**34 (26%) 8.76**	6 (5%) −0.50	7 (5%) −2.36
**PGO**	56 (39%) −0.69	39 (27%) −1.02	4 (3%) −1.75	**27 (19%) 6.64**	17 (12%) −0.30
**PGU**	265 (38%) −2.18	251 (36%) 1.61	38 (5%) −1.19	32 (5%) −1.27	**120 (17%) 3.14**

***Note:*** Values in parentheses were adjusted residuals. Highlighted cells showed statistical significance, α<0.01

**Table 5:** Observed frequencies (conditional probabilities) and adjusted residuals in technology-centered style.

**Initial behavior**	**Response behavior**				
	**PGD**	**PGT**	**PGC**	**PGO**	**PGU**
**DGP**	**529 (63%) 15.24**	20 (2%) −12.22	16 (2%) −6.54	18 (2%) −3.21	258 (31%) −2.66
**DGT**	231 (27%) −12.09	**237 (28%) 16.90**	24 (3%) −5.00	23 (3%) −2.01	**326 (39%) 3.87**
**DGC**	28 (24%) −4.33	2 (2%) −3.79	**61 (52%) 21.78**	1 (1%) −1.69	25 (21%) −2.97
**DGO**	14 (20%) −4.06	1 (1%) −2.99	7 (10%) 1.42	**28 (39%) 16.17**	21 (30%) −0.80
**DGU**	54 (47%) 0.94	2 (2%) −3.72	10 (9%) 1.31	4 (4%) −0.13	44 (39%) 1.07
	**DGP**	**DGT**	**DGC**	**DGO**	**DGU**
**PGD**	364 (38%) −7.12	**417 (43%) 6.00**	53 (6%) 0.36	46 (5%) 1.75	79 (8%) 0.87
**PGT**	**198 (77%) 10.75**	35 (14%) −8.24	7 (3%) −2.00	10 (4%) −0.09	6 (2%) −3.45
**PGC**	40 (40%) −1.26	24 (24%) −2.73	**23 (23%) 8.06**	4 (4%) −0.00	9 (9%) 0.50
**PGO**	31 (52%) 0.88	16 (27%) −1.66	1 (2%) −1.29	**6 (10%) 2.41**	6 (10%) 0.68
**PGU**	265 (46%) 0.12	225 (39%) 1.48	20 (3%) −2.34	12 (2%) −2.77	50 (9%) 1.11

***Note:*** Values in parentheses were adjusted residuals. Highlighted cells showed statistical significance, α<0.01
